# Effects of Applying Gamified Exercise in Health Education Classes on Physical Activity Levels in School-Aged Children: A Randomized Controlled Pilot Study

**DOI:** 10.1097/jnr.0000000000000746

**Published:** 2026-05-26

**Authors:** Li-Chun HSIAO, Chi-Jane WANG, Ya-Ping YANG, Jong-Ni LIN

**Affiliations:** 1 Department of Nursing, College of Medicine, National Cheng Kung University, Tainan, Taiwan; 2 Department of Nursing, National Tainan Junior College of Nursing, Tainan, Taiwan; 3 Department of Post-Baccalaureate Nursing, and Department of Nursing, Da-Yeh University, Changhua, Taiwan; † Contributed equally

**Keywords:** school-age children, physical activity, inclusive curriculum, gamification, multidisciplinary

## Abstract

**Background:**

Declining physical activity (PA) among schoolchildren is a global issue. Schools play a crucial role in children’s PA, as they spend most of their waking hours at school. Therefore, developing an easy-to-implement school-based physical activity program is important.

**Purpose:**

This study was designed to evaluate the effectiveness of using gamified exercise in health education classes to enhance PA levels among schoolchildren.

**Methods:**

A randomized controlled pilot design was applied. Two elementary schools were randomly assigned, respectively, to an experimental group (EG; *n* = 47) that received a gamified fitness program with reciprocal physical education and a control group (CG; *n* = 44) that received reciprocal physical education only. The gamified fitness program, developed and validated by a multidisciplinary team, was delivered during health education classes. PA levels were measured using AI-wireless wrist sensors worn during schooltime at three points, including the first week (T1), sixth week (T2), and 12th week (T3) of the program. Program feasibility was assessed using participant satisfaction and analyzed using Generalized Estimating Equations (GEE).

**Results:**

PA levels at the three time points (T3-T1) were significantly different between the two groups. The results of GEE analysis revealed that daily steps (mean difference ± *SE*: 4,068.4 ± 1,144.1 steps), total PA (84.4 ± 16.0 min), light PA (LPA; 72.6 ± 15.3 min), and moderate to vigorous PA (MVPA; 12.1 ± 2.9 min) increased significantly more over time in the EG than the CG. Moreover, approximately 85% of the EG identified the PA program as being satisfactory and feasible.

**Conclusions/Implications for Practice:**

The results of the pilot trial conducted in this study demonstrate the feasibility of integrating gamified exercise into school health education classes and the potential of this program to increase physical activity levels in schoolchildren significantly. The success of the developed program is attributable to its multidisciplinary approach, engaging design, and focus on core strength and cardiorespiratory endurance. Gamified fitness in health education may be used to promote activity, support inclusive policies, and reduce disparities among school-age students.

## Introduction

Physical activity (PA), essential for the overall development of school-aged children, has been shown to benefit their physical health, mental well-being, and academic performance (Melby et al., 2021). However, despite these recognized benefits, many children, especially adolescents aged 11–17, fail to meet recommended PA levels (Sedumedi et al., 2021). Taiwanese children and adolescents also face a high prevalence of physical inactivity (Wu & Chang, 2019), despite policies mandating 150 min of weekly moderate to vigorous PA (MVPA) in primary schools (Weaver et al., 2021). Achieving adequate PA levels in adolescents is a significant global public health issue. Therefore, increasing physically active time outside of physical education (PE) classes is considered critical to improving overall PA levels in children.

### Solutions and Strategies for Improving Physical Activity in Children

Several related studies have proposed solutions and strategies for improving PA in children. These include integrating PA programs into academic subjects such as mathematics (Vetter et al., 2020) or PE (Kliziene et al., 2021) courses. Also, strategies such as implementing Classroom Active Breaks (i.e., the CABs model; Gallè et al., 2020) and providing short PA sessions before and after school (Sacheck et al., 2021) have been proposed. However, the evidence regarding the effectiveness of these strategies in improving PA is inconsistent, and the abovementioned strategies do not consider how to sustain the beneficial effects through, for example, incorporating related activities into non-PE classes such as health education (HE) to increase active time and achieve sufficient PA levels. Therefore, further exploration is needed to develop innovative solutions, create suitable implementation opportunities, and maintain program execution.

### Gamification

Gamification refers to the application of game-design elements in nongame contexts to influence behavior, motivation, and engagement (Koivisto & Hamari, 2019; Krath et al., 2021). In the context of education, gamification involves incorporating elements such as points, badges, leaderboards, levels, and challenges into teaching and learning activities to enhance students’ experiences (Smiderle et al., 2020). These game mechanics may impact psychological engagement in learners positively by promoting enjoyment, autonomy, and social interaction (Ryan & Deci, 2020).

In recent years, gamification has been increasingly integrated into teaching models across various educational domains for children and adolescents as a strategy to promote PA and improve motivation (Mo et al., 2024; Santos et al., 2023). A systematic review of 19 studies concluded gamification to be an effective approach to increasing motivation in adolescents during PE courses, with reported benefits including enhanced autonomy, improved social skills, and a more positive classroom environment (Sal-de-Rellán et al., 2025). Effective strategies are often grounded in the principles of gamification, which include creating engaging and meaningful learning environments, offering rewards, supporting goal achievement, and fostering enjoyment (Aznar-Ballesta & Vernetta, 2023; Vasconcellos et al., 2020).

Most of the gamification strategies related to promoting physical activity have already been applied in primary school physical education to promote students’ motivation and commitment (Arufe-Giráldez et al., 2022; González-González et al., 2018).

### Study Purpose

This study was designed to examine the effects of a gamified PA program integrated into HE classes on PA levels and program satisfaction in schoolchildren.

### Hypotheses

1. Participants in the gamified PA program will show more significant improvements in daily steps, total PA (TPA), light PA (LPA), and moderate to vigorous PA (MVPA) than their control group peers.
2. Participants in the gamified PA program will self-report a high level of satisfaction with the developed program.

## Methods

### Study Design

Using a pilot randomized controlled trial conducted between February and May 2021, the effectiveness of a gamified PA program in improving active time and levels was assessed in an HE class setting.

### Sample Size Calculation

The minimum sample size necessary to achieve an α = .05, power = .80, and effect size = 0.1 with an *F*-test (repeated measures, within-between interaction), 2 groups, 3 measurements, assumed correlation of repeated measures = .5, and nonsphericity correction *ε* = 1 was calculated using G-Power 3.1 software.

A priori power analysis was conducted to estimate a feasible sample size under practical constraints rather than justifying post hoc significance only. The small effect size (Cohen’s *f* = 0.10) was based on Cohen (1988) and Serdar et al. (2020), with recruitment limited by school policy to two classes per school. Anticipating a 20% loss in follow-up, a minimum sample of 90 participants was calculated.

### Study Participants

#### Participant Eligibility

All of the students enrolled in the selected sixth-grade classes (typical class size ≈ 25; age 11–12 years) were eligible to participate in the study. Exclusion criteria were (a) having a physical disability that limited participation in MVPA and (b) not providing written parental consent.

#### Sampling and Group Assignment

**Figure 1:**
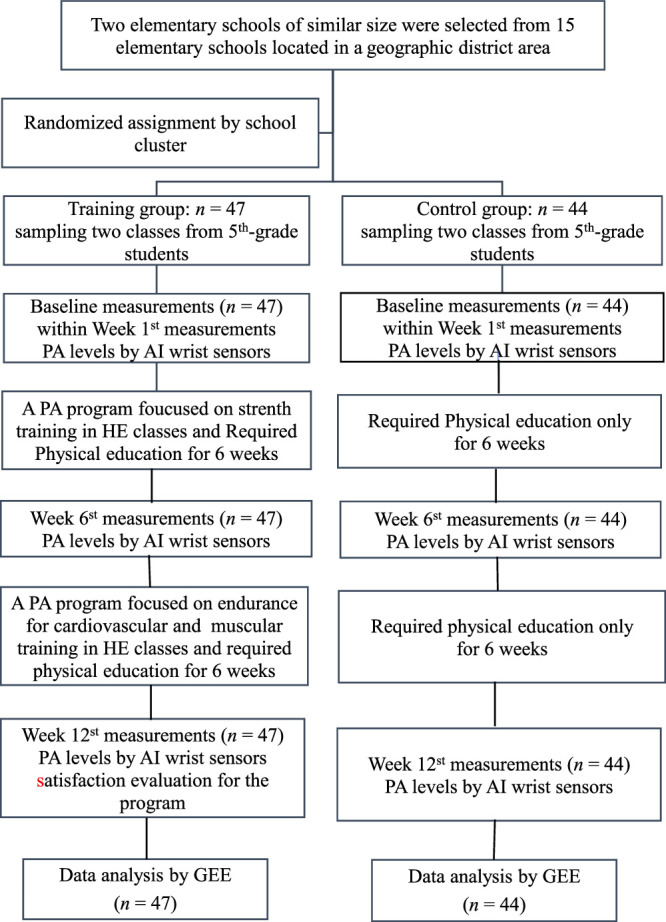
Recruitment of Participants Flowchart *Note.* PA = physical activity; AI = artificial intelligent; HE = health education; GEE = Generalized Estimating Equations.

Two public elementary schools of similar size and student composition located in the same urban district of southern Taiwan were approached after obtaining approval from each principal. From each school, two sixth-grade classes were chosen at random, yielding four intact classes. All eligible students in the selected classes were invited to participate. Cluster randomization was then applied at the school level, with one school allocated to the experimental group and the other allocated to the control group (Figure 1). Both groups continued participating in their respective compulsory PE curricula. The experimental group additionally participated in the developed PA program.

### Ethical Consideration

Approval from the Cheng Kung University Human Ethics institutional review board (No IRB#: E-AR-106-441) was received before data collection and recorded on the clinical trial website. The principal, teachers, students, and their parents were informed about the aims and procedures of this study. All of the data were kept confidential and used only for academic research purposes. Consent was obtained from the participants, and written parental consent was obtained before data collection.

### Compulsory Physical Education

​Both the EG and CG participated in comparable PE classes, adhering to Taiwan’s 12-Year Basic Education Curriculum Guidelines. These guidelines recommend two 40-min PE sessions per week, totaling 80 min and focusing on activities that enhance flexibility, endurance, and muscular strength (Table 1). For students aged 11–12, suggested exercises include butterfly stretches, walking lunges, long-distance running, dancing, cycling, arm circles, and cat-cow poses.

**Table 1:** The Physical Activity in School in the Experimental and Control Groups

Group	School Setting	Instructor	Content	Activities/Tasks	Duration (per Session)	Frequency (per Week)	PA in School Duration (per Week)	Total PA Duration (per Week)
EG	PE classes	PE teacher	School curriculum	• Flexibility exercise • Endurance training • Stretching exercise	40 min	Twice a week for 16 weeks	80 min	150 min
	HE classes	Major: fitness instructor Minor: class teacher and school nurse	Warm-up	• Fast-paced side-stepping • Arm swings • Lunges • Squats	5 min	Once a week for 12 weeks	40 min	
			Main exercise	• Strengthening exercise: the training focus is in weeks 1st to 4th	30 min			
				• Cardiovascular endurance: the training focus is in weeks 5th to 8th				
				• Muscular endurance: the training focus is in weeks 9th to 12th				
			Cool-down	• Upper body stretches • Seated forward bend • Knee-to-chest pose • Hamstring stretch	5 min			
	Classes break	School nurses	Active play	• Higher-intensity level of PA with team-based competition	10 min	Triple a week for 12 weeks	30 min	
				• By students’ preference, such as playing ball, fitness walking, the Eagle-grabs-chicks game				
CG	PE classes	PE teacher	School curriculum	• Flexibility exercise • Endurance training • Stretching exercise	40 min	Twice a week for 16 weeks	80 min	80 min

*Note.* EG = experimental group; CG = control group; PE = physical education; HE = health education; PA = physical activity.

#### Intervention

The school-based PA program for the EG was developed in three stages: evidence-based fitness training design, multidisciplinary planning, and the integration of gamification elements. The final version of the intervention was integrated into the health education curriculum (Table 1).

#### Evidence-based Fitness Training Design

A systematic literature review (2015–2021) across Embase, MEDLINE, Cochrane CENTRAL, and CINAHL resulted in 1,078 articles, from which 22 relevant school-based interventions were identified. Drawing on these studies, the program focused on core strength, cardiorespiratory endurance, and muscular endurance through age-appropriate exercises such as planks, running games, and body-weight movements. Activities were designed to be safe, engaging, and progressively challenging for the participants, aiming to improve physical fitness, concentration, and overall health (Kliziene et al., 2021; Neil-Sztramko et al., 2021; Sacheck et al., 2021; Weaver et al., 2021).

#### Multidisciplinary Collaboration to Ensure Intervention Feasibility

To ensure feasibility, the program was co-developed in cooperation with a multidisciplinary school team, including administrators, teachers, school nurses, and support staff. This team participated in workshops to align goals and plan implementation steps and to address scheduling, resource use, and evaluation issues.

#### Gamification Strategy Design

To increase motivation, the program included gamification elements, including point systems (one point per completed task), team competitions, digital badges, and short-term rewards over a 2-week cycle. Student progress and achievements were shared via a closed Facebook group to enhance engagement and peer support.

### Procedures

The school-based PA intervention was implemented in coordination with the existing HE curriculum and class schedule, and consisted of the following three main components:

#### Scheduling and Duration

HE classes were scheduled twice weekly over a 20-week semester. Each class was 40–50 min in length. The gamified PA program was delivered once a week for 40 min over a period of 12 weeks. This schedule was designed to fit within academic constraints and is supported by previous research on the effectiveness of similar interventions. In addition, 10-min active play sessions were conducted during class breaks three times per week, for a total of 30 min of these sessions per week. Thus, students engaged in approximately 150 min of physical activity per week, which includes 70 min of the PA program and 80 min of compulsory PE classes (Table 1).

#### Fitness Training Implementation

Three structured fitness sessions targeting core strength, cardiorespiratory endurance, and muscular endurance were implemented in the HE classes (Appendix I, Supplemental Digital Content 1, http://links.lww.com/JNR/A9). Each session followed a standardized format including a 5-min warm-up, main activities, and a 5-min cool-down. The activities were age-appropriate and designed to ensure safety, engagement, and progressive development.

#### Active Play Sessions

In addition to HE-based training, classroom teachers conducted 10-min active play sessions during breaks three times weekly. These sessions included various physical activities based on expert recommendations, and allowed flexibility for teacher-led adaptation. School nurses supported the sessions by monitoring student safety and encouraging participation.

### Instruments

#### Demographic Characteristics and Physical Health Indicators

Participant demographic data, including age, gender, and height, were obtained from official school records. Physical health indicators, including weight, body mass index (BMI), and body fat percentage, were measured at baseline (T1) using a multifrequency bioelectrical impedance analysis device (IOI 353). The IOI 353 employs a validated protocol to provide accurate and reliable assessments of body composition.

#### Physical Activity Levels

PA levels—including TPA, LPA, MVPA, and daily step counts—were measured objectively using a noncommercial, university-developed wearable sensor, which integrates a triaxial accelerometer and neural-network-based algorithms to classify activity types and estimate energy expenditure. LPA was defined as 1.5–3.0 metabolic equivalents (METs), and MVPA as > 3.0 METs. Gait-related parameters, including step counts and walking distance, were derived using gait phase detection techniques. Classification accuracies of greater than 95% for activity type and energy estimation, up to 98.9% for walking pattern classification, and 96.4% for walking distance estimation have been previously reported, confirming the high validity and reliability of this device when used in school-based PA monitoring (Lin et al., 2012; Wang et al., 2012).

#### Satisfaction With the Physical Activity Program

Participant satisfaction with the PA program was evaluated across five domains: (a) integration within health education classes, (b) training content, (c) application of gamification elements, (d) activity opportunities during breaks, and (e) perceived effectiveness in enhancing PA levels. Satisfaction was measured using a five-point Likert scale ranging from “*very dissatisfied*” to “*very satisfied*.” In addition, qualitative feedback was collected via open-ended questions regarding fitness training, scheduling, implementation, and duration, with this feedback intended to provide a reference to guide future program refinement.

The satisfaction scale was reviewed by six experts to ensure content validity and tested with ten students for reliability, showing Cronbach’s alpha values between .80 and .90, indicating good to excellent consistency.

### Data Collection

Physical health indicators were assessed at baseline (T1) using the IOI 353 multifrequency bioelectrical impedance analyzer, with measurements calculated automatically and verified by the research team.

Physical activity data were collected using a validated, custom-designed wearable sensor at three time points, namely at baseline (T1), week 6 (T2), and week 12 (T3). All of the participants in the EG wore these sensors from 8:00 a.m. to 4:00 p.m. for 8 hr per day over five consecutive school days during each measurement period. At the end of each school day, these participants returned the devices to their teachers, who regularly assisted with daily collection. Sensors were checked and recharged as needed to ensure consistent data recording. If any participant had more than 2 days of missing data within a measurement period, their data for that time point were excluded from analysis.

Satisfaction data were collected from the EG at the end of the 12-week program using a structured self-administered questionnaire, supplemented by qualitative feedback obtained through group discussions and participant comments hosted on a dedicated Facebook platform.

### Data Analysis

The collected data were analyzed using IBM SPSS Statistics 25.0 (IBM Corp., Armonk, NY, USA). Descriptive statistics were used to present the distribution of all variables, including percentage, mean, and standard deviation (*SD*). Considering the confounders within the two groups, the χ^2^ test for categorical variables and the *t* test for continuous variables were applied to identify potential between-group differences in demographics or pretest data and then adjusted these as covariates. The Generalized Estimating Equation (GEE) was used to determine the effect of the intervention program on PA.

## Results

### Demographic Characteristics Between the EG and the CG

Ninety-one participants completed this study (47 in the EG and 44 in the CG). The participants had a mean age of 11.4 years (*SD* = 0.5). Over half were boys (*n* = 49, 53.8%), and 45 (49.4%) were overweight or obese. The mean body mass index (BMI) and body fat were 20.9 kg/m^2^ (*SD* = 4.5) and 21.2% (*SD* = 7.2), respectively. In terms of the PA data, the mean of daily steps, TPA, LPA, and MVPA were 8,205.1 (*SD* = 3,694.4) steps, 130.9 (*SD* = 106.0) min, 111.5 (*SD* = 107.4) min, and 18.9 (*SD* = 13.9) min, respectively. Regarding PA outcomes between the EG and the CG, significant differences were identified in terms of daily TPA and LPA in the baseline assessments (Table 2).

**Table 2:** Between-Group Comparison of Demographic Characteristics

Characteristic	Total (*N* = 91)	EG (*n* = 47)	CG (*n* = 44)	*t* or χ^*2*^	*p*
	*n* (%)	*n* (%)	*n* (%)		
Gender				0.09	.772
Female	42 (49.2)	21 (44.7)	21 (47.7)		
Male	49 (53.8)	26 (55.4)	23 (52.3)		
Body size				0.17	.921
Underweight ^a^	7 (7.7)	4 (8.5)	3 (6.8)		
Normal ^b^	32 (35.2)	17 (42.6)	15 (34.1)		
Overweight/obese ^c^	45 (49.4)	26 (48.9)	19 (43.2)		
Missing	7 (7.7)		7 (15.9)		

	**Mean (*SD*)**	**Mean (*SD*)**	**Mean (*SD*)**		
Body mass index (BMI; Kg/m^2^)	20.9 (4.5)	21.4 (4.9)	20.3 (3.7)	3.6	.301
Body fat (%)	21.2 (7.2)	21.4 (8.1)	21.1 (6.1)	3.1	.854
Age (y)	11.4 (0.5)	11.4 (0.5)	11.5 (0.5)	0.5	.371
Daily steps	8,205.1 (3,694.4)	7,639.2 (2,6630.7)	8,558.9 (2,710.4)	1.6	.128
Daily TPA (min)	130.9 (106.0)	160.9 (91.9)	104.2 (41.4)	−3.8	.001
Daily LPA (min)	111.5 (107.4)	141.8 (95.6)	78.6 (40.8)	−4.1	.001
Daily MVPA (min)	18.9 (13.9)	19.1 (17.1)	25.6 (13.9)	1.9	.072

*Note.* EG = experimental group; CG = control group; TPA = total physical activity; LPA = light physical activity; MVPA = moderate to vigorous physical activity.

^a^ Female: BMI < 14.9 kg/m^2^; male: BMI < 15.0 kg/m^2^. ^b^ Female: 14.9 kg/m^2^ ≤ BMI<20.9 kg/m^2^; male: 15.0 kg/m^2^ ≤ BMI<21.0 kg/m^2^. ^c^ Female: BMI ≥ 20.9 kg/m^2^; male: BMI ≥ 21.0 kg/m^2^.

### Comparison of PA Levels Between the EG and CG Over Time

The results of the GEE analysis after adjusting for variables, including BMI, TPA, and LPA, for PA levels at T1, T2, and T3 for the EG and CG are discussed below, with the effects of the interaction between group and time in T2-T1 and T3-T1 shown in Table 3.

**Table 3:** The GEE Analysis-Adjusted Model Tests the Changes in Physical Activity (PA) Levels Within and Between Groups Across Different Times

PA Level	T1	T2	T3	T2-T1		T3-T2		T3-T1	
(EG = 47 vs. CG = 44)	Mean (*SE*)	Mean (*SE*)	Mean (*SE*)	Diff. (*SE*)	*p*	Diff. (*SE*)	*p*	Diff. (*SE*)	*p*
Daily steps									
EG	7,931.8 (263.3)	9,513.6 (366.8)	9,836.6 (1,061.8)	1,581.8 (380.6)	.001	323.1 (951.3)	.730	1,904.8 (1,019.6)	.061
CG	8,259.7 (305.2)	7,506.1 (349.4)	6,096.1 (406.3)	−7,53.6 (386.65)	.050	−1,410.0 (376.5)	< .001	−2,163.6 (521.1)	.001
Diff. (*SE*)	−327.9 (424.6)	2,007.4 (522.9)	3,740.5 (1,190.7)	2,335.3 (543.3)	.001	1,733.1 (1,021.9)	.090	4,068.4 (1,144.1)	.001
Daily total PA (min)									
EG	128.9 (2.7)	175.0 (10.6)	144.9 (13.2)	46.1 (11.8)	.001	−30.1 (14.2)	.040	15.9 (14.3)	.262
CG	135.3 (2.5)	130.5 (5.9)	66.9 (7.9)	-4.8 (5.7)	.402	−63.6 (8.3)	<.001	−68.4 (7.3)	.001
Diff. (*SE*)	−6.3 (4.6)	44.6 (11.4)	78.1 (15.3)	50.8 (13.1)	.001	33.5 (16.5)	.040	84.4 (16.0)	.001
Daily light PA (min)									
EG	107.6 (2.1)	152.4 (10.2)	127.5 (12.9)	44.8 (11.3)	.001	−24.9 (13.1)	.060	19.9 (13.6)	.142
CG	112.1 (2.1)	109.9 (5.8)	59.4 (8.1)	−2.2 (5.2)	.682	−50.6 (8.3)	<.001	−52.7 (7.2)	.001
Diff. (*SE*)	−4.5 (3.9)	42.5 (10.9)	68.1 (15.2)	46.9 (12.4)	.001	25.6 (15.5)	.100	72.6 (15.3)	.001
Daily MVPA (min)									
EG	21.3 (1.0)	22.6 (1.7)	17.6 (1.4)	1.9 (2.1)	.383	−4.9 (1.6)	<.001	−3.7 (2.1)	.083
CG	23.4 (0.9)	20.7 (1.2)	7.6 (1.4)	−2.7 (1.6)	.117	-13.1 (1.6)	<.001	−15.8 (2.1)	.001
Diff. (*SE*)	−2.1 (1.4)	1.9 (2.1)	10.0 (1.9)	3.9 (2.6)	.141	8.2 (2.2)	<.001	12.1 (2.9)	.001

*Note.* CG = control group; Diff. = difference within the group and between groups; EG = experimental group; T1 = baseline; T2 = sixth week; T3 = 12th week; *SE* = standard error; each PA indicator was adjusted for sex, baseline BMI, baseline light PA, and baseline MVPA (moderate to vigorous PA).

#### Between-group Comparison of Daily Steps Performance

In the EG, the average daily steps were 7,931.8 ± 263.3 at T1, 9,513.6 ± 366.8 at T2, and at 9,836.6 ± 1,061.8 at T3. The increase in step counts in the EG across the time points was significantly larger than that observed in the CG, which increased by 2,335.3 ± 543.3 between T1 and T2 and by 4,068.4 ± 1,144.1 between T1 and T3. Although the number of steps between T2 and T3 was higher in the EG, the intergroup difference with the CG was not statistically significant. Nevertheless, the EG showed a greater increase than CG in daily steps across the three time points over the 12 weeks.

#### Between-Group Comparison of Daily TPA Performance

TPA increased by 46.1 ± 11.8 min between T1 and T2 and by 15.9 ± 14.3 min between T1 and T3 in the EG, but declined by 30.1 min between T2 and T3. In the CG, TPA decreased across all three time points. Overall, TPA was significantly higher in the EG than the CG at both T2 and T3.

#### Between-Group Comparison of Daily LPA Performance

In the EG, LPA increased by 44.8 ± 11.3 min between T1 and T2 and by 19.9 ± 13.6 min between T1 and T3, but declined by 24.9 ± 13.1 min between T2 and T3. In the CG, LPA decreased across all three time points. While the LPA values for both groups were similar at T3, the increases in LP between T2 and T3 and T1 and T3, respectively, were both more significant in the EG than the CG.

#### Between-group Comparison of Daily MVPA Performance

In the EG, MVPA did not significantly increase between T1 and T2, and significantly decreased between both T2-T3 and T1-T3. In the CG, MVPA declined significantly across all three time points. However, the change in MVPA was significantly higher in EG than in CG for both T2-T3 (8.2 ± 2.2; *p* < .001) and T1-T3 (12.1 ± 2.9; *p* < .001). This indicates that over the 12 weeks, the MVPA level was consistently higher in the EG than the CG, even though the CG had reported relatively higher MVPA levels at T1.

### Satisfaction With PA Program Participation During HE Class

#### Quantitative Evaluations Using a Structured Self-Administered Questionnaire

Regarding participant satisfaction with the PA program, approximately 80%–90% of EG participants expressed satisfaction with the evaluated aspects, including the integration of physical training into HE classes, the content of the physical training, the application of gamification strategies in training, and the active play during class breaks. In terms of the evaluated aspects, the highest satisfaction rate (90%) was observed for the application of gamification strategies and active play during class breaks. The effectiveness of the Gamified Health Fitness Training in improving PA also received high satisfaction ratings, with 88% of participants in the EG expressing either “very satisfied” or “satisfied.”

#### Qualitative Feedback

In terms of the qualitative feedback related to the PA program and its implementation, a majority of the participants expressed that “The fitness training is incredibly enjoyable; the competitive aspect kept us engaged and motivated to participate in these activities.” Some also revealed, “If classes were designed this interesting, I would love to go to school and learn every day.” However, some expressed that the training time was too short and hoped it could be extended. Furthermore, some mentioned that certain postures were too challenging to implement, especially those related to core muscle training.

## Discussion

### Main Findings

The findings of this randomized controlled pilot study provide evidence that integrating a gamified PA program into HE classes significantly enhanced PA levels among the 11- to 12-year-old study participants. The primary findings demonstrate the EG displayed noteworthy enhancements across all levels of PA (daily steps, TPA, LPA, and MVPA) from the initial week to the 12th week (T1-T3). Conversely, the CG showed a decrease in all PA levels over the 12-week period.

### The Effectiveness of the Program in Enhancing PA Levels

The PA program integrated into the HE curriculum in this study had an enhancement effect on daily step numbers at T3 compared with both baseline (T1) and CG, similar to outcomes observed in previous related interventional studies (Greier et al., 2020; Neil-Sztramko et al., 2021). Compared with the CG, the EG engaged in 70 additional minutes of higher-intensity PA per week, achieving 150 total minutes weekly (vs. 80 for the CG). This is consistent with the World Health Organization’s recommendation for accumulated PA in children and adolescents aged 5–17 years.

Moreover, the number of daily steps for the CG in this study decreased significantly over time, which differs from previous studies. However, according to normative data from the Canada Physical Activity Guide, among children aged 6 to 11 years, boys average 12,000 to 16,000 steps/day while girls average 10,000 to 13,000 steps/day (Neil-Sztramko et al., 2021). In this pilot study, the daily steps of EG participants significantly increased from 7,931.8 (±263.3) at baseline to 9,836.6 (±1,061.8) after the 12-week PA program, which did not meet the recommended daily steps in the abovementioned guidelines for children 6–11 years old. Potential reasons for this disparity include variations in training duration and the necessity to offer participants more education and motivation to embrace a healthy and active lifestyle. Addressing the challenge of ensuring daily steps meet the recommended levels for children will be a significant consideration in the planned subsequent large-scale study aimed at program refinement.

In this study, TPA, LPA, and MVPA increased at T2 (sixth week) but decreased at T3 (12th week). This decrease was likely attributable to increased academic demands near the semester’s end. This trend was observed in both the EG and CG. However, the decrease in the EG was smaller than in the CG. In future studies, applying a longer follow-up period may better assess the long-term effects of the intervention.

The findings of this study at T2 are consistent with Vetter et al. (2020), which also reported positive effects on PA, and the overall effects (T1-T3), with significant increases for each level of PA in the EG compared with the CG, are consistent with other studies (Gallè et al., 2020; Have et al., 2018; Gutiérrez-Martínez et al., 2018; Neil-Sztramko et al., 2021) that implemented interventions for more than 10 weeks and incorporated similar and repetitive exercises during required lessons or class break times.

However, the effects declined in both groups between T2 and T3. During this period, it was possible that the participants were busy preparing for final examinations, which may have reduced the time available for engaging in physical activity. Another possible reason relates to the training regimen. In this study, the training regimen involved focusing on one major exercise during each of the two 3-week periods to optimize training efficacy. According to the Physical Activity Guidelines for Children 5–12, children should participate in a variety of age-appropriate physical activities designed to achieve optimal health, wellness, fitness, and performance benefits (Weaver et al., 2021).

Therefore, future research should focus on investigating available strategies, policy changes, and the appropriate components of fitness regimens as potential approaches to achieve substantial effects on higher levels of physical activity for children.

Regarding the MVPA, the average value increased during the first 6 weeks (T1-T2) in the EG only and decreased in both groups over the second 6 weeks (T2-T3). This finding is similar to a systematic review that concluded from 33 studies that school-based physical activity interventions likely result in minimal or no increase in time spent in moderate to vigorous physical activity (Neil-Sztramko et al., 2021). Even though evidence suggests that multicomponent interventions that include physical activity during the school day (e.g., active lessons and breaks) can strongly increase time spent in MVPA (Bernal et al., 2020). In the literature, elementary school students are generally recommended a target of doing 60 min of regular exercise per day (Weaver et al., 2021). Although school-based physical activity interventions may improve physical fitness, a focus on higher-intensity activities is needed.

Although the PA intervention was not found to significantly improve TPA, LPA, and MVPA, which was contrary to expectations, it is noteworthy that all PA levels declined significantly in the CG across all time periods. This suggests the gamified exercise program implemented in this study favorably influences PA levels in school settings. Subsequent research into related fitness training programs should explore feasible strategies for maintaining their positive effects even when the program duration extends beyond 6 weeks. Ensuring the continuity and effectiveness of a PA program over an extended period is crucial in maximizing its impact on PA and overall fitness levels.

### Gamification and Multidisciplinary Teams in the PA Program

Gamification in primary school physical education has been shown to improve the motivation and commitment of students to physical activity. In the context of active video games, gamification has been shown to increase motivation among young people to participate in physical exercise and adopt health-promoting behaviors (Arufe-Giráldez et al., 2022; González-González et al., 2018). The PA training program applied in this study integrated gamification and active play during class breaks, achieving a high satisfaction rate (90%). This approach positively impacted and enhanced PA levels at school, possibly due to clear goals, team-based competitions, points, virtual rewards, and badges motivating fitness training involvement, which are frequently used in education to increase motivation and interest in learners (Gallè et al., 2020). The use of gamification as a feasible strategy appears to positively impact student engagement and enthusiasm in terms of class participation and PA performance. Integrating Self-Determination Theory (Sotos-Martinez et al., 2024) into gamified physical activity programs can enhance participant motivation by fulfilling basic psychological needs such as autonomy, competence, and relatedness, leading to improved adherence and increased physical activity levels in daily life. Research indicates that when these needs are satisfied, individuals are more likely to engage in and maintain physical activity behaviors.

Due to their potential to capture a wide range of expertise and viewpoints, multidisciplinary teams are frequently employed in designing multicomponent physical activity interventions for children in school or clinical settings (Mooses et al., 2021; Weaver et al., 2021). According to the findings of related articles, the members of these teams often include educators, physical fitness experts, school staff, and relevant issue experts (Morell-Azanza et al., 2019). In this study, school nurses were enlisted to assist class teachers in enhancing children’s awareness of PA levels, devising gamified strategies, and ensuring the safety of children during active play. School nurses, pivotal in promoting the health and well-being of children in primary schools, handle a range of responsibilities tailored to the unique needs of elementary-aged children (Pawils et al., 2023). Incorporating school nurses within a multidisciplinary team is an effective strategy for enhancing PA levels in students.

### Strengths and Limitations

This study has several strengths and limitations. Its main strength is its school-based, multidisciplinary, and collaborative teaching approach that involves nursing, education, and physical education professionals. The gamified physical training program was integrated into the existing HE curriculum, and school resources and support from key staff were leveraged to make it a cost-effective and sustainable intervention. Sports coaches led the fitness training in the HE classes. Meanwhile, gamification strategies were applied to increase student engagement. The program also provides a valuable reference for developing active learning courses in physical education and includes repeated measures to track long-term effectiveness.

The several limitations of this study include its short duration (12 weeks) and small implementation scale, which limit the generalizability of findings. The original 300-min course (Kim et al., 2020) was shortened to 150 min due to school schedule constraints, and participants were limited to children 11–12 years old. Future research using a longer duration and large sample is recommended to support policy development and wider applicability. This study was conducted in two schools in Taiwan, providing valuable insights into the implementation of gamified physical activity programs. However, the applicability of these findings to different educational and cultural contexts requires careful consideration for future studies.

### Implications for School Health Policy, Practice, and Equity

The effectiveness of the PA program implemented in this study may be attributed to factors such as the utilization of a multidisciplinary team, HE class-based design, gamified activities and its focus on core strength and cardiorespiratory endurance. This PA program may provide valuable evidence for school health managers and educational policymakers when developing customized PA programs for implementation in school settings.

Furthermore, research has shown that single mothers with children tend to be more inactive than partnered mothers, highlighting the importance of providing appropriate social support programs to reduce disparities in PA participation (Chao et al., 2024). These findings underscore the need for school-based PA programs to consider family and role-related constraints when designing inclusive strategies that promote equity in physical activity engagement.

### Conclusions

The results of this study indicate that integrating a 12-week gamified physical fitness program in classroom settings is a feasible approach to effectively promoting children’s physical activity levels in school. This gamified program, implemented during elective courses, positively impacted daily steps and overall physical activity by involving multidisciplinary teams and focusing on core strength and cardiorespiratory endurance. This approach may serve as a valuable reference for school health managers and education policymakers in designing and implementing customized physical activity programs in school settings.

## Supplementary Material
