# Supplementary material for: Effects of Applying Gamified Exercise in Health Education Classes on Physical Activity Levels in School-Aged Children: A Randomized Controlled Pilot Study
Source: J Nurs Res. 2026 May 26;34(3):e461. doi: 10.1097/jnr.0000000000000746 (PMC13200866; doi:10.1097/jnr.0000000000000746)
Supplement: Supplementary file 1 [file jnr-34-e461-s001.docx]

**Appendix I**

12-Week Fitness Training Program Overview

| Topic | Week | Content | Extended Exercise |
| --- | --- | --- | --- |
| Core strength | 1^st^ | Hand plank, crunch, paired exercise (cycling), exercise in small groups (butt walking in a straight line) | 1. Hand plank: 30 seconds 2. Crunch: 20 times |
|  | 2^nd^ | Elbow plank, side plank, reverse crunch, paired exercise (butt walking with a peer in a circle), exercise in small groups (shift plank) | - 1. Elbow plank: 30 seconds   2. Side plank: 20 seconds (right and left each)   3. Mountain climber: 20 times (right and left each) |
|  | 3^rd^ | Shift plank, rotation side plank, grouping exercise (shift plank, ball pass—legs, ball pass—hands and legs) | 1. Shift plank (hand to elbow): 5 times (right and left each) 2. Rotation side plank: 15 times (right and left each) 3. Reverse crunch: 20 times |
|  | 4^th^ | Cycling, side crunch, exercise in small groups (standing bug, ball pass—V-hand and legs) | 1. Cycling: 30 times (right and left each) 2. Side crunch: 30 times (right and left each) 3. Hand plank: 40 seconds |
| Cardio-respiratory endurance | 5^th^ | Static warm-up, aerobic dance, stretching | 1. Burpee: 20 times 2. Running in place: 20 times (right and left each) 3. Shuffle steps 3 steps and shuttle run: 10 times (right and left each) |
|  | 6^th^ | Static warm-up, aerobic dance, exercise with triangular pyramids, stretching | 1. Vertical jump: 15 times 2. Lateral jump: 30 times (right and left each) 3. Speed running in place: 20 seconds |
|  | 7^th^ | Dynamic warm-up, fitball, stretching | 1. Running track (brisk walking in the straightaway and slow walking in the curve): 2 laps 2. Jumping lunge: 20 times (right and left each) 3. Mountain climber: 20 times (right and left each) |
|  | 8^th^ | Dynamic warm-up, fitball, exercise with triangular pyramids, stretching | 1. Running track (sprinting in the straightaway and jogging in the curve): 2 laps 2. Jumping Jack: 20 times 3. Step jump: 10 times (forward, backward, right, and left count as 1 time) |
| Muscular endurance | 9^th^ | Static warm-up, basic muscular training of the upper limbs and lower limbs | 1. Squat: 20 times 2. Lunge: 15 times (right and left each) 3. Knee-down push-up: 10 times (right and left each) |
|  | 10^th^ | Dynamic warm-up, basic muscular training of the upper limbs and lower limbs | 1. Squat jump: 20 times 2. Push-ups: 15 times 3. Sumo squat: 30 times (right and left each) |
|  | 11^th^ | Dynamic warm-up, advanced muscular training of the lower limbs | 1. Lunge jump: 15 times (right and left each) 2. Split lunge: 10 times (right and left each) 3. Bear crawl: 10 times |
|  | 12^th^ | Dynamic warm-up, advanced muscular training of the lower limbs | 1. Pull up: 10 times 2. Triceps dip: 10 times 3. Biceps curl: 10 times |
